# From twisting to settling down as a nurse in China: a qualitative study of the commitment to nursing as a career

**DOI:** 10.1186/s12912-020-00479-x

**Published:** 2020-09-12

**Authors:** Jiao Ye, Aimei Mao, Jialin Wang, Chizimuzo T. C. Okoli, Yuan Zhang, Huiqiong Shuai, Min Lin, Bo Chen, Linli Zhuang

**Affiliations:** 1 People’s Hospital of Yubei District of Chongqing City, Jianshe Ave, Chongqing, China; 2 School of Nursing, Chengdu University of Traditional Chinese Medicine, Shierqiao Ave, Chengdu, Sichuan China; 3 Kiang Wu Nursing College of Macau, Est. Repouso No.35, R/C, Macau, China; 4 University of Kentucky College of Nursing, BREATHE 315 College of Nursing Building, Lexington, KY 40536-0232 USA

**Keywords:** Clinical nurses, Career commitment, Qualitative research, China

## Abstract

**Background:**

The nurse workforce shortage, partially caused by high work turnover, is an important factor influencing the quality of patient care. Because previous studies concerning Chinese nurse work turnover were predominantly quantitative, they lacked insight into the challenges faced by nurses as they transition from university to their career. A successful transition can result in new nurses’ commitment to the career. As such, this study sought to understand how new nurses commit to the career, and focused on identifying facilitators and barriers to such commitment.

**Methods:**

This was a qualitative study using a grounded theory design. Through purposive sampling, clinical nurses were recruited from hospitals in Western China to participate in semi-structured interviews. The data was analyzed through coding to develop categories and themes.

**Results:**

Theoretical saturation was achieved after interviewing 25 participants. The data revealed the ‘zigzag journey’ of committing to the nursing career. The emerging core theme was “getting settled”, indicating that new nurses needed to acclimate to the work reality in the nursing career. By analyzing the data provided by the participants, the researchers concluded that the journey to getting settled in nursing compassed four stages:1) “sailing out with mixed feelings”, 2) “contemplating to leave”, 3) “struggling to stay”, and 4) “accepting the role”. For most participants, nursing was described as a way to earn a living for their family, not as a career about which they felt passionate.

**Conclusions:**

Committing to the nursing career is a complicated long-term process. There seems to be a lack of passion for nursing among the Chinese clinical nurses participating in this study. Thus, the nurses may need continued support at different career stages to enhance their ability to remain a nurse for more than economic reasons.

## Background

The shortage of nurses is a major problem faced by almost all countries worldwide. Despite the increased enrollment of nursing students in the past two decades, China is still far from having a sufficiently staffed nursing workforce. By the end of 2016, the total number of registered nurses in China was 3.507 million, and the number of nurses per thousand population was 2.54 [1]. This is a large proportion gap compared with the 9 to 17 per thousand population rates observed in developed countries in Europe and North America [2]. It is estimated that 2 to 3 million.

more nurses in China are needed for the current service supply [1].

In addition to insufficient enrollment in nursing schools, high turnover rates are another contributor to the nursing shortage in China. In 2007, the Ministry of Health of China conducted a national survey in 696 tertiary hospitals, and reported that the average turnover rate was 5.8% [3]. More recent studies reported turnover rates between 6.5 and 20.0% [4–7], indicating that the condition is worsening. The continuous turnover of nurses not only wastes time and money for both nursing schools and health care institutions, but also results in a decreased quality of health services [8, 9].

In China, previous studies have identified various factors contributing to turnover in the nursing workforce, such as job dissatisfaction, burnout/fatigue, troubled relationships with peers and clients, weak professional passion, etc. [4, 5, 10]. These predominately quantitative studies revealed the multifaceted nature of the decision making processes among nurses who decide to leave nursing. However, quantitative studies are limited as they can only be used for large-scale investigations or predictions at a macro level [11]. Additionally, quantitative studies provide general information about a social phenomena at a fixed point in time while the dynamics, individual differences, and the integrity of the phenomena are easily overlooked [11, 12]. Previous studies lack the subtle description of the internal struggles of nurses who make decisions to remain a nurse. Such a decision making process is a process of developing a commitment to nursing as a career. Career commitment refers to the degree of unwillingness to change a career due to an individual’s recognition, emotion and investment in the career; it is the ‘psychological contract’ signed by an individual and professionals [13]. Quantitative studies in China have failed to capture the process dynamics of career commitment [14–18]. Qualitative approaches may better examine human phenomena in the context of social sciences. Moreover qualitative approaches can clarify ambiguous or unknown areas of research, and may provide a better way to describe fundamental social processes in life [19]. Among them, the grounded theory is an approach that attaches importance to the dynamic development of processes [19]. Therefore, grounded theory was selected for the present study to describe the development process for clinical nurses committing to nursing as a career.

At present, there are no qualitative studies from China about career commitment and nursing. Previous studies have mainly focused on the process of nurses’ professional identity development, and the research subjects were mainly novice nurses. Professional identity refers to the process in which nurses recognize their professional roles, meet the competency requirements of their roles and form a clear career commitment [20]. Conceptually, professional identity establishment is part of developing career commitment. New employees are most vulnerable regarding retention during the first few months of their employment [21], when they are most likely to encounter difficulties in adapting to their new professional roles [22]. Several qualitative studies [23–25] have described the development of professional identity among novice nurses.

A person’s professional identity is often initiated during formal education, and develops further after entering the workplace. Newly graduated students often find that their initial education may conflict with the value of the working world, and this conflict may cause them to feel depressed because they cannot control the reality. Kramer [23] first called this feeling “Reality Shock”. Ellis [24] described the choices of nurses when they encountered reality shock. Some nurses switch to other careers because they are disappointed with the nursing career. Others try to change jobs or return to school to find a better alternative. A few others persist in pursuing nursing in accordance with their initial ideals, but complain about the system in which they work. Kramer [23] divided the transition process of newly graduated nurses into several stages: honeymoon, disorientation and disillusionment, recovery and balance. Duchscher [25] also described a step by step process whereby newly graduated nurses became competent as: doing, being, and knowing. The ‘doing’ stage usually occurred 3 to 4 months post-graduation, the ‘being’ phase occurred 6 to 8 months post-graduation, and the ‘knowing’ stage occurred 1 year after graduation. The studies mentioned above all describe the dynamic process of identity development of the novice nurses. However, they focused on the transition period of new nurses in the first one or 2 years post-graduation. Although these studies provided some reference for the development of nurses’ career commitment, it is important to note that professional identity and career commitment are two different concepts. Moreover, developing career commitment is a lifelong process. There is currently a lack of in-depth knowledge on how long-term practices influence the development of career commitment.

The development of the nursing career in China has been a dramatic history. The baccalaureate nursing program was initiated in 1920, but was replaced in 1950 by a three-year diploma program, which was run by hospitals or vocational schools. After the bachelorette program was reinitiated in 1983, nursing education has flourished in China [26]. However, nursing education in China remains complex with various levels of programs including the vocational school-based or college-based diploma programs, and the university-based baccalaureate and postgraduate (master or doctorate) programs. New graduates from different programs are eligible to become registered nurses as long as they pass the national registration licensing test.

The objectives of this study were to describe the process from which clinical nurses in China commit to nursing as a career. The study was driven by the following questions:

1. What is the process of career commitment development among nurses in China?
2. What factors influence the developmental process of nurses’ career commitment?
3. What are the critical points during the process of career commitment when nurses decide to change careers?

The findings from this study have important implications for the development of measures to enhance career commitment among nurses and, thus, reduce their chances of leaving the career.

## Methods

### Design

Grounded theory was used for this study to identify the commitment process through the constant comparison technique [27]. Grounded theory attaches importance to understanding the dynamic processes that often result in the development of a theory. However, the ultimate goal of this study was to describe the process of clinical nurses committing to nursing as a career in China to determine the various factors that affect the process and gain more insight into the phenomenon of nurse turnover.

### Participants

The study was conducted in Chengdu, the capital city of Sichuan Province in Southwestern China. Purposive [28] and snowball [29] sampling were used to recruit the participants. Registered nurses working full-time in a health care institution for at least 1 year were eligible to participate.

The various backgrounds of the potential participants, such as gender, length of service, working unit, duty styles (day or night shift), etc., were taken into account during recruitment to gain insight to the identity development of nurses from different aspects. The primary researcher was a postgraduate nursing student who was on rotation in different wards in a large hospital during data collection. She invited the potential participants to take part in the study and asked schoolmates, colleagues, and recruited participants to recommend potential participants from other hospitals. Chengdu has 72,400 registered nurses. Therefore, sufficient potential participants were available and the researcher could choose the most suitable participants for the research objectives.

In grounded theory, theoretical sampling is used to identify participants who can provide data to confirm, clarify, and expand the emerging themes [27]. In this study, the later participants could respond, clarify and supplement the emerging ideas that had been conceptualized in the interviews of the previous participants. Based on grounded theory, when researchers have defined, checked, and explained the relationships and a range of variation within and between analytical categories they reach ‘theoretical saturation’ and can terminate sampling [11].

### Data collection

From December 2016 to May 2017, semi-structured interviews were conducted to collect data. The primary researcher (the first author) developed the interview guideline (See Additional file 1 for details) by reviewing the literature and consulting with the research team. At the beginning of the interviews, general questions were asked, including “How did you make the choice to be a nurse?” or “What are your experiences being a nurse?” As the conversations proceeded, the questions became more specific. The researcher adjusted the order of questions, the form of asking, and the topic of questions based on a specific situation. For example, if a respondent was unclear about a question or hesitated to respond to a question about the career, they were asked, “What makes you less sure about the nursing career?”, “What would you do if you had another chance?” and “What is your future plan?” The interviews were scheduled on the mutual convenience of the participants and the researcher in terms of time and place. Eleven interviews were conducted in the on-duty rooms in the wards where the participants worked, six in the teaching rooms in the wards, five in the home of the participants, two in the dormitory room of the researcher, and one in a coffee shop. The interviews lasted approximately 30 to 60 min. All the interviews were recorded under the agreement of the participants.

### Data analysis

Strauss and Corblin (1990) suggested that data collection and analysis should be.

simultaneous. All the recorded interviews were manually transcribed verbatim by the researcher shortly after the interviews. Grounded theory coding techniques [30] were used to analyze the data,and included: 1) reading the original materials carefully to obtain a comprehensive understanding of the data, 2) developing the first-level coding as ‘open coding’ in which the researcher examined the data using line-by-line or incident-by-incident coding to define actions and perspectives of the participants related to their remaining in or leaving nursing, 3) developing the second-level coding as ‘axial coding’ in which the goal was to discover and establish the various relationships between the generic conceptions (for example, the relationship between the social images of the nurses and low pay), 4) developing the third-level coding as ‘theoretical coding’, referring to the systematic analysis of all discovered generic conceptions to obtain one or more “core conceptions”, and 5) clustering clues of the story by reading all the interview data by reconstructing themes and categorizing them in a certain order while interspersing the researcher’s insights and reflections. Constant comparison was used in the whole analytical process by exploring similarities and differences in participants’ narratives by comparing data with data, data with categories, and categories with categories. Thus, the concepts were refined and the process was made explicit [27].

### Ethics considerations

The research was approved by a university ethics committee (Chengdu University of Traditional Chinese Medicine Reference no. 2018KL-076). All the potential participants were provided information on the objectives and procedures of the research. A signed consent was obtained from the participants before the interviews. The participants were informed about their rights to withdraw from the study whenever they felt uncomfortable. A code was assigned to each participant to ensure anonymity and all the data from the participants were accessible only to the research team.

## Results

In this study, saturation was achieved by the seventeenth interview, but eight additional interviews were collected to ensure consistency in the data. The study comprised 25 nurses from eight hospitals: 21 females and 4 males, aged between 22 and 52 years. Detailed information about the participants’ basic information, including age, education, marital status, service areas, length of service, and duty styles, is provided in Table 1.

**Table 1:** Demographics of the study participants

Variable	Number
Age (years), mean ± SD	30.5 ± 8.0 (22–52)
Gender	
Male	4
Female	21
Levels of nursing education	
Vocational school-based or college-based diploma	8
Bachelor’s degree	14
Master’s degree or higher	3
Marital status	
Unmarried	12
Married	13
Working unit	
Cardiology department	5
Gynecology department	3
Digestive department of endocrinology	2
General surgery department	2
Nephrology department	2
Operating room	2
Proctology department	2
Pediatric department	2
ICU	2
Other departments	3
Duty style	
Daytime work only	11
Daytime and night shifts	14
The length of employment	
< 3 years	8
≥ 3and < 5 years	6
≥ 5 and < 10 years	4
≥ 10 years	7

*SD* standard deviation

**Fig. 1:**
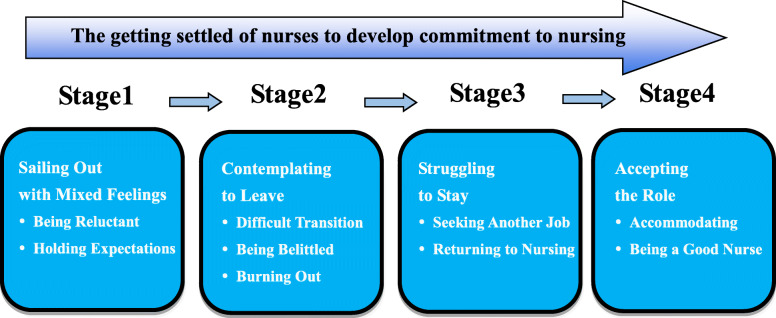
Journey of the nurses to develop commitment to nursing. Describes the four stages of the journey of nurses to develop commitment to nursing and the relationships of the categories and subcategories involved in each stage. The journey to settling into nursing encompassed several stages: sailing out with mixed feelings, contemplating to leave, struggling to stay, and accepting the role

In grounded theory, the resultant themes are interpreted and conceptualized into core categories [27]. In this study the core theme, “getting settled”, captured the dynamic nature of the nurses’ struggle in decision making to remain in nursing. The journey to settling into nursing encompassed several stages: sailing out with mixed feelings, contemplating to leave, struggling to stay, and accepting the role. Although some participants undergo the journey linearly, others undergo the process forwards and backwards. For example, an experienced nurse who had accepted her role as a nurse might contemplate leaving the career again after she was issued a complaint note by her patient. Two or three experienced nurse participants never considered leaving the career. Figure 1 describes the four stages of the journey and relationships of the categories and subcategories involved in each stage. We have provided interpretative explanations of the categories and subcategories in detail in the following sections.

### Sailing out with mixed feelings

This stage describes the journey of establishing a professional identity, post-gradation, that was initiated before entering the nursing practical world. Two subcategories were identified related to this first stage: ‘being reluctant’ and ‘holding expectations’.

This first stage reflected the mixed feelings of the new nurses when they initially.

joined nursing.

#### Being reluctant

Most of the participants (18 of 25) elaborated on the long-term rigid image of nursing as a job of low pay, low social status, high risk and heavy workload. They recalled that joining nursing school was not their own decision when they were applying for a college after high school graduation. Some of them were persuaded by those around them to join a nursing school, while others were transferred to nursing by admission officers after they had failed their applications for other majors.

A20 was one of the few participants who joined a nursing school willingly. She initially began medical school but transferred to nursing in the second year of her college studies. She made the transfer because she knew that nursing graduates could find a job more easily than their medical school counterparts. A20 came from the countryside. She considered that after graduation, she could secure a job as a nurse in an urban hospital to leave her rural environment. Although she stayed in Chengdu, the capital city, after graduation she came to realize that she had chosen a job she did not truly like.

Despite being disappointed with nursing, the participants felt compelled to enter.

nursing once they graduated because nursing was a very specific career. A1 indicated her reluctance to enter nursing when she graduated, *“I did not like nursing at the time I graduated. However, I didn’t know what else I could do. The four years’ training only developed my nursing skills rather than other talents.”*

#### Holding expectations

Many participants (20 of 25) expressed that they developed a certain degree of appreciation for nursing because they knew more about nursing during nursing school, *“Before I joined the nursing school, I thought that all nurses could do was an IM* (intramuscular injection) *and an IV* (intravenous injection)*. People said that all nurses did was to take care of patients, as your mother takes care of you at home. As I learned more in the nursing school, I realized that the way nurses take care of patients is not the way your mother takes care of you. Nurses do their work based on systematic mastery of health-related knowledge and clinical skills.”* A1.

Thus, the nurses’ understanding of nursing was quite different from that of the public. According to the new graduates, nursing was a career and they could showcase the ‘professionalism’ of nursing in their future work.

Nursing was also regarded as a stable job through which nurses could help their families in terms of both financial and health care support. Some of the participants remembered their expectations of their new jobs when they graduated.

*At the time we graduated, we were clear that compared with other jobs, nursing was hard. Nurses had heavy workloads and they were not paid decently. However, nursing graduates could find a job more easily. Nursing was a stable job.* A24.

*When I graduated and joined clinical nursing, I had little expectation for my new role.*

*Nursing knowledge and skills could be a help to my family and friends when they approached me for health advice*. A14.

Getting a stable job immediately after graduation was very important to the newly graduated nurses. They could obtain the first job in their lives and make contributions to their family, implying the formal establishment of their adulthood.

### Contemplating to leave

While the newly graduated nurses expected to enter practice to showcase their learning achievements, their limited passion for nursing was not enhanced by their fresh experiences. They found a huge gap between the image of nurses they had constructed during their studies and what they observed inside the wards. They.

became disappointed and frustrated. Their disappointment was due to negative aspects like “difficult transition”, “being belittled” and “burning out.”

#### Difficult transition

The participants all faced difficulties immediately after entering the clinical world. They were required to be on rotation and work in different wards. The required rotation time in every ward lasted from one or 2 weeks to several months. It was common that, when the neophytes had become familiar with the routines of one ward it was the time for them to rotate to the next ward. The neophytes were given too many tasks and responsibilities before they felt ready. In addition to the heavy workloads, the nurses had too many shifts and received a low salary. A15 described his difficult time when he was on ward rotation, *“After I worked for some time I felt tired, lots of night shifts, too many tasks and too low salary. I felt hopeless for the future.”*

The participants also complained that they were unfairly treated during the transition time, as A7, who had worked as a nurse for 2 years said, *“It is quite normal that a novice does not know everything. You’ve got to go from unknowing to knowing. But some of the experienced nurses and even the head nurses did not understand that. They would reprimand you if you did not do things the way they had expected. They thought that you should be able to do everything once you had graduated. You were inferior if you did not.”* A7 was a talented student in her nursing school and held several notable positions in class and school levels. However, discouraged by her new colleagues, she intended to leave nursing soon after she became a clinical nurse.

A11 spoke of being unfairly held accountable by her new colleagues too. *“Because we were neophytes, we became the suspects when something wrong happened in the ward. Sometimes we were already judged accountable even prior to investigation.”A11.*

#### Being belittled

Contrary to the respectable image of nurses as ‘angels in white,’ which had been ingrained by nursing school teachers, the participants observed a largely negative attitude towards nurses and nursing from society.

*After I had worked for several years I found something about nursing was quite different from what I had expected. When the patients were recovered there were always the doctors’ names on the “Thank you” note. There was no mention of nurses. When the patients were unsatisfied with their doctors, they overtly expressed anger to nurses, rather than to their doctors. We became the scapegoats. At one time, I looked down upon myself. I was shamed to tell others that I was a nurse. I felt that nursing was a lowly regarded work.* A2.

A2 is now the head-nurse of a proctologic ward. She had been a nurse for 32 years. She confessed that she had lost interest in nursing ‘now and again’ during her career. She once intended to join a part-time medical school and then work as a doctor in her uncle’s hospital after graduation. She did not go to the school when she realized that she could not go to her uncle’s hospital. A2’s case suggests that clinical nurses’ commitment to nursing may be contextual. There seemed to be higher or lower levels of commitment throughout their career.

The belittled image of nurses was reflected in the patients’ references to nurses, as the nurses elaborated that they were called by patients as ‘little servant’, ‘little nurse’ and ‘waitress’*.* They were not only belittled by the patients but also looked down upon by the doctors and administrators of the hospitals with whom they worked.

*Not just patients. The whole medical system lacks respect for nurses. In our hospital, all the doctors, the head of the ward* (it was the doctors that were the heads) *all look down upon nurses, although we are in the multidisciplinary team.* A7.

*The doctors look down on nurses. They think that nurses do not have a speciality. They are only an middleman between patients and doctors, telling doctors what their patients asked and then telling the patients what their doctors suggested. The nurses know nothing. They only do IM and IV under doctors’ orders.* A3.

#### Burning out

All the participants emphasized the heavy workload imposed on nurses. One participant elaborated that after she had finished rotations and was assigned to a ward, she had to work several nightshifts and was expected to manage a heavy workload independently. The patients in her ward were severely ill and she was under immense pressure to manage the entire ward. Other participants echoed that, in addition to a heavy workload in the wards, new nurses had to take several examinations required by the hospitals or wards, as A16 said, *“We just started but they regarded that we could manage all. Additionally , neophytes had to take on lots of tests, paper based and skill exams. We just did not have time to prepare work and the tests. We were exhausted.”*

### Struggling to stay

Nurses who wanted to leave nursing began to seek or even try other jobs. Some eventually left nursing, but most did not succeed in the process. They either could not find another job or returned to nursing after having tried other jobs. There are two subcategories in this stage, ‘seeking another job’ and ‘returning to nursing’.

#### Seeking another job

Because the nursing career is highly specialized, nurses encountered many obstacles in finding new jobs. The difficult process made them realize that they could not do anything but nursing.

*I really didn’t want to continue nursing at that time … Before 2013, nurses could take part in the national postgraduate entrance exam of clinical medicine, I took the exam twice, but failed both. Then I did not try anything else. I felt that I had no ability to do anything except nursing.* A22.

A22 wanted to change from nursing to medicine because her husband, who was a doctor in the same hospital with her, complained that being a nurse “can neither make money nor take care of the family”. She had to stay in nursing after her failed attempts to become a doctor.

A8, a male, also talked about his failure in changing jobs, *“I started looking for other jobs at that time, but it did not go well. I had considered jobs in enterprises and companies. I prefer freedom but I found that no jobs provided me the expected freedom.”*

Over time, new nurses passed the transition period and became more familiar with their work. They were also awarded higher salaries once they had successfully completed the required rotation time. The new nurses’ determination to leave nursing gradually shrank. A8 was assigned to a ward after a three-years’ rotation. He was not as firm in changing job as he had been 3 years before, *“My rotation was almost over, and the salary was not as low as before. Meanwhile, I had gotten familiar with the work, so I hesitated. I didn’t know whether to go or stay.”*

The participants were clear that quitting nursing might imply unemployment before they found another job. In certain circumstances this could impose a risk to the economic stability of their family. A22 got married shortly after she graduated and soon had her first child. The arrival of the child increased her family’s financial burden, which forced her to drop the quitting intention, *“W*e *had a child, and we needed money to raise him. I had to seriously consider what would happen to my family if I quit nursing. I could not afford the risk. I decided to stop looking for other jobs.”* For participants like A22, their salaries from nursing really mattered to their family’s survival.

#### Returning to nursing

The decision to leave nursing was not only related to personal career satisfaction but also to family living standards. The nurses had to balance the economic benefits of nursing and other jobs. Some of them who had tried other jobs decided to return to nursing because of the better economic prospects of nursing.

A15 is a male nurse and he returned to nursing after he had worked for some time as a salesperson, a similar experience as another male nurse participant A8, *“After I quit nursing, I joined a company and did marketing. My experiences made me realize that the sales work was not so good and the nursing work was not so bad. At the same time people around me told me that under the current situation, doing nursing would be more stable and better than doing marketing.”* (A15). He eventually returned to nursing after he had worked for half a year as a salesman and was in his fourth year as a nurse.

### Accepting the role

After experiencing a long period of struggle to leave nursing, the nurses finally accepted their role as nurses. A feeling of having settled down was prominent at this stage. They used such words as “not floated” “no more other jobs” “acceptance of nursing” and “no other thoughts” to describe their calmness. There are two subcategories in this stage: “accommodating” and “being a good nurse”.

#### Accommodating

The participants knew that the difficulties they had experienced did not exist only in their wards or hospitals but were common challenges facing the career at large. Many participants took this problem rationally. They eliminated their own resistance to the nursing career through self-adjustment, thus adapting to the professional environment and accepting the role of nurses. A2 described her psychological adjustment process, “ *There is no correct and comprehensive evaluation of nurses by the public. We can not change this situation, but we can adjust our mindset to accept the role of nurses and reduce the resistance to career. When you help the patient solve the problem, you can appreciate yourself. You can never ask others to appreciate you, but you can appreciate yourself.*”

The participants also realized that, as neophytes, they had somehow exacerbated the shortcomings of nursing. They needed to look at nursing more comprehensively. A17 described her changed views on nursing during different stages of her career, *“Before I joined nursing I didn’t know that I would do nightshifts nor did I know that nursing was so hard. I was disappointed with the situation I was in when I was a neophyte. I struggled and was unhappy. Later, I realized that I had to earn a living by doing nursing. Once I had decided to be in nursing in my lifetime, I thought that, instead of complaining, it should be better for me to accept both advantages and disadvantages of a job. Nursing has many disadvantages, but it has merit*s, too.*”*

The nurses found that once they stopped complaining and saw nursing from another angle, nursing was a worthy career. Like A17, A25 stated that she was no longer pessimistic about nursing as she had been previously. Once she ‘changed the color of the glasses through which she viewed nursing’, she found a different image of nurses. *“Recently, I found that most patients were more respectful to nurses than before. Nurses now do a lot of health education for patients … the nursing work is not as mechanical as before, and nurses can do more than in the previous time. You know, many hospitals have opened nursing clinics.”* Her accounts indicated that nursing.

was gaining more autonomy; therefore, nurses were gaining more respect.

#### Being a good nurse

Most of the nurses decided to stay in nursing not because they were passionate, but because nursing was a good way to support their family. They emphasized that, regardless of whether they loved nursing they would wholeheartedly carry out a nurse’s responsibilities each day they were on duty.

A8 once looked for other jobs and now decided, at least temporarily, to stay in nursing. He was determined to be a good nurse, despite not loving nursing. *“In the future I may still leave nursing if there is a better job for me. On the other hand, as long as I am a nurse, I should conscientiously carry out nurses’ responsibilities. I do not love nursing. However, I will feel uneasy if I do something tarnishing nurse’s image.”*

There were several nurses who felt re-energized once they decided to stay. For example, A25 was currently a part time postgraduate nursing student. She decided to pursue a Master’s degree in nursing because a higher degree meant a better prospect for career development. A17 expressed her ambition to make some achievements in her career field. She expected to be promoted to be the deputy head nurse in her ward within the next 2 years. She was now applying for a hospital sponsored research project.

## Discussion

This is the first qualitative study in China examining the clinical nurses’ journey of developing a commitment to nursing as a career from graduation to several years of clinical practice. The findings mirror previous findings on professional identity development. For example, novice nurses tend to feel confused about their career at the beginning of their employment [22]; salary, workload and social status are important factors affecting nurses’ professional identity and separation decision-making [31]. Although nurses complained of nursing as a career with low pay and low social status, they eventually found, particularly after having tried other jobs, that other jobs were difficult for nurses. They also learned that no job was easy, so people should cherish the jobs they had. There were competing factors that influenced the nurses’ decision, including low pay vs. available employment, low social status vs. career stability, autonomy vs. control, and idealism vs. pragmatism.

Previous studies have revealed the difficulties that neophytes face in adapting to new roles of a career. The findings have varied in the duration of the difficult times last, ranging from half a year to 2 years [23–25, 32]. The neophytes were susceptible to leaving their jobs during such transition times. This study supports these previous findings,implying that the transition time is largely influenced by the working arrangements carried out by the institutions in which neophytes are employed. The study found that the longer is the rotation time, the longer is the transition time, because neophytes were required to become familiar with each of the wards through which they rotated. Since an increasing number of hospitals in China prefer nurses with abroad knowledge of a variety of wards, they are requiring new nurses to rotate through more wards, which may result in an increased length of transition time [33].

The worrying factor is that the negative social image of nursing was, according to the participants, co-constructed by the whole health care system: the patients, doctors, and administrators, indicating that the whole system has failed nurses. The prospect of job stability is an attraction to nursing students. It is important to note that, while students from low socio-economic backgrounds, such as rural China, may be more likely to choose nursing to eliminate poverty, nursing may become a career comprising people of low socio-economic status. This composition may further lower the social status of nursing [34, 35].

This study further supports assertions that nursing commitment development is an endless process [36]. There is not a clear beginning nor end point. The participants in the study traced their dissatisfaction with nursing to the pre-college time. A qualitative study with those who quit nursing in China also found that ex-nurses were not fond of nursing when they were recruited by nursing schools of different educatioan levels [37]. Although some nurses in our study tried to advance their career development after they had decided to settle down, others acknowledged no passion for nursing. They were termed as “passive staying” in Zhu’s study on ex-nurses [37]. They may again become vulnerable to leaving under unfavourable conditions or better employment opportunities.

Development of career commitment is culturally framed [38–40]. For example, one study from Japan found the deep influences of Confucianism, collectivism, and power distance on clinical nurses’ perceptions and practices [39]. The study added evidence from China that the development of career commitment may be culturally constructed. A strong familial influence was evident in selecting and remaining in nursing among the study participants. The participants repeatedly provided accounts that family members played important roles in their decision to join and stay in nursing. Their choice to remain in nursing was also largely influenced by family economic conditions or family members’ attitudes towards nursing. In other countries, due to different cultural backgrounds, differences exist in the employment motivation of nurses. A qualitative study from Iran found that clinical nurses attributed much of their motivation to their spiritual beliefs [41] The general importance of spirituality and subsequent attention to moral aspects of the job among the Iranian population has brought about consideration of a broader perspective beyond the material and formal aspects of nursing considered in other locations [42]. This finding may be related to Iran being a highly religious Muslim country. The current status of nursing in India relates to the intersections of religion, caste and gender through India’s colonial history [43]. With its association with menial work, nursing has traditionally been viewed as a ‘polluted’ occupation that presents a threat to the social identity of those from the upper Hindu castes;thus, it is an inappropriate choice of employment for them [44].

### Relevance to clinical practice

The present study suggests that nurses harbor feelings of ‘unfairness’ over the inferiority of nurses’ status among the multidimensional healthcare team. Nurses were regarded as assistants to doctors, not their partners, as they should be. This was related to the lack of autonomy of nursing as a legitimate career. In fact, lack of authority and autonomy has long been an obstacle preventing nursing from standing out as a full career [38, 45]. There is a need for nursing scholars in China to work with those overseas to explore the boundary of nursing responsibilities and ways to gain authority and autonomy for nursing.

The study revealed a “blame” culture in China that contributed to neophytes’ intention to leave nursing. This culture victimised the neophytes more than the experienced nurses. A friendlier environment towards new employees needs to be created and may include appropriate supervision for new nurses and clear guidance for their responsibilities.

Helping new nurses make long-term career plans may be another way to enhance their sense of belonging and reduce their tendency to leave the career [46]. While support should be provided for neophytes, experienced nurses also need inspiration, because they may become unstable in their nursing career if an unfavourable event occurs. Continued support for nurses at various career stages seems necessary. For experienced nurses who have decided to continue nursing and are carrying out their career development plan, they need support from nursing administrators for their short- and long-term career goals. They may be assigned some teaching and management responsibilities to enhance their sense of fulfilment.

### Limitations

Some of the interviewees in this study were the primary researcher’s colleagues during her clinical rotation time. They might not have provided sufficient or truthful information because of concern over the revelation of personal information. Additionally, most of the participants in this study were clinical nurses in public hospitals above Grade-A in Chengdu, a large metropolitan city. Clinical nurses in smaller hospitals or cities may have different experiences from those at urban large hospitals. Therefore, the findings of the study cannot be generalized.

## Conclusion

The study portrayed the difficult journey of clinical nurses in China in cultivating a commitment to nursing. A dynamic process was described as nurses searched for the meaning of nursing in their daily working life. The process was affected by many factors, which served as facilitators or barriers to nurses’ intention to remain a nurse. Although the study divided the journey linearly in four stages, there were no strict boundaries among the stages. The findings of the study added new knowledge to the enhancement of career commitment among nurses in the Chinese context.

While nursing educators, researchers and administrators can learn from the study the importance of a supportive environment for nurses in various stages of career development, the wider structural system should play a more important role to change the overall unfavorable environment for nurses in China. Only when the long-term problems facing nursing, such as low pay, low social status, and heavy workload are addressed can nursing become a truly attractive career to prospective and current nurses.

## Supplementary information

**Additional file 1.** Interview Outline.

## Data Availability

The datasets generated and analysed during the current study are not publicly available to ensure data confidentiality, but are available from the corresponding author on reasonable request and with the consent of the research participants.

## Abbreviations

TCM: Traditional chinese medicine
SD: Standard deviation
