# Supplementary material for: From twisting to settling down as a nurse in China: a qualitative study of the commitment to nursing as a career
Source: BMC Nurs. 2020 Sep 12;19:85. doi: 10.1186/s12912-020-00479-x (PMC7488578; doi:10.1186/s12912-020-00479-x)
Supplement: Supplementary file 1 — Additional file 1. Interview Outline. [file 12912_2020_479_MOESM1_ESM.doc]

**Additional file 1**

**Interview Outline**

1. How did you make the choice to be a nurse?

2. What are your experiences being a nurse?

3. What is your usual working state?

4. What emotions do you often experience in work? When do you experience these emotions?

5. During the years of nursing work, have you ever thought of resigning? What makes you less sure about the nursing career? (Further questioning based on the respondents' answers)

6. What would you do if you had another chance?

7. What is your future plan?
